# From hands‐on to remote: Moderators of response to a novel self‐management telehealth programme during the COVID‐19 pandemic

**DOI:** 10.1002/ejp.1968

**Published:** 2022-05-24

**Authors:** Carlos Gevers‐Montoro, Zoha Deldar, Andrea Furlan, Eric A. Lazar, Erfan Ghalibaf, Arantxa Ortega‐De Mues, Ali Khatibi

**Affiliations:** ^1^ Madrid College of Chiropractic – RCU María Cristina Madrid Spain; ^2^ Department of Anatomy Université du Québec à Trois‐Rivières Trois‐Rivières Quebec Canada; ^3^ Department of Psychology McGill University Montreal Quebec Canada; ^4^ KITE, Toronto Rehabilitation Institute University of Toronto University Health Network Institute for Work & Health Toronto Ontario Canada; ^5^ Institute for Cognitive Science Studies (ICSS) Tehran Iran; ^6^ Centre of Precision Rehabilitation for Spinal Pain University of Birmingham Birmingham UK; ^7^ Centre for Human Brain Health University of Birmingham Birmingham UK

## Abstract

**Background:**

In March 2020, state‐wide lockdowns were declared in many countries, including Spain. Citizens were confined to their homes and remotely supported activities were prioritized as an alternative to in‐person interactions. Previous data suggest that remote and self‐management interventions may be successful at reducing pain and related psychological variables. However, individual factors influencing the effectiveness of these interventions remain to be identified. We aimed to investigate the psychological and motivational factors moderating changes in pain observed in chiropractic patients undertaking a novel telehealth self‐management programme.

**Methods:**

A cohort of 208 patients from a chiropractic teaching clinic was recruited to participate in the study. Patients received telehealth consultations and individualized self‐management strategies tailored for their current complaint. They were encouraged to make use of these strategies daily for 2–4 weeks, whilst rating their pain intensity, motivation and adherence. Validated questionnaires were completed online to assess catastrophizing, kinesiophobia and anxiety.

**Results:**

A total of 168 patients completed the first 2 weeks of the programme, experiencing significant reductions in all variables. Kinesiophobia emerged as a key factor influencing pain reduction and moderating the association between motivation and pain relief. In turn, adherence to the programme was associated with lower pain intensity, although moderated by the degree of motivation.

**Conclusions:**

In the context of COVID‐19, when introducing remote and self‐management strategies, pain cognitions and motivational factors should be taken into consideration to foster adherence and yield better pain outcomes.

## 1 INTRODUCTION

In March 2020, a large part of the world shut down after the World Health Organization declared the coronavirus disease 2019 (COVID‐19) a pandemic (Mahase, 2020). Social distancing was the main public health intervention employed to mitigate the impact of COVID‐19 (Islam et al., 2020; Lewnard & Lo, 2020; Wilder‐Smith & Freedman, 2020), leading countries such as Spain to declare state‐wide confinements (Government of Spain, 2020). Social distancing measures and the pandemic have been linked to social isolation, physical inactivity (Ammar et al., 2020; Tison et al., 2020) and psychological distress (Garcia‐Alvarez et al., 2020; Ozamiz‐Etxebarria et al., 2020; Rodriguez‐Rey et al., 2020), which can all contribute to ongoing pain (Alzahrani et al., 2019; Hammig, 2019; Joseph et al., 2020). As a result, the pre‐existent burden of pain conditions was expected to increase (Clauw et al., 2020; Thacker & Mansfield, 2020).

COVID‐19 lockdowns limited access to healthcare services, carrying detrimental consequences for patients’ outcomes (Gevers‐Montoro et al., 2022; Nieto et al., 2020). In Spain, severe restrictions were imposed on in‐person services (Carrillo‐de‐la‐Pena et al., 2021), including the closure of chiropractic clinics (@QuiropracticAEQ, 2020). Whilst chiropractors typically rely on manual therapy for the management of musculoskeletal conditions, exercise prescription and patient education are also part of routine care (Beliveau et al., 2017; Clijsters et al., 2014). These do not require physical presence and may be alternatively provided as part of remote care.

With widespread community transmission, remote services took precedence over in‐person interactions, including health encounters (Cohen et al., 2020; Henriquez et al., 2020). As a consequence, an adaptation towards telehealth services for non‐life‐threatening conditions was witnessed early in the pandemic (Eccleston et al., 2020; Green et al., 2020; Lynch et al., 2020; Puntillo et al., 2020). Previous data showed that telehealth interventions, heavily relying on self‐management and exercise, are beneficial in reducing pain, disability (Adamse et al., 2018; Dias et al., 2021) and associated psychological symptoms (Cavanagh et al., 2019; Gannon et al., 2019; O'Brien et al., 2018). However, the effectiveness of such programmes is strongly influenced by adherence, motivation and fear‐avoidance beliefs (Ackerman et al., 2013; Nicholas et al., 2012; Söderlund & von Heideken Wågert, 2021). Understanding barriers and facilitators for adhering to remote self‐management interventions is essential to improve their effectiveness (Fernandes et al., 2021; Svendsen et al., 2020). Yet, the role of these factors to ensure the adaptation of healthcare services to social distancing in the COVID‐19 framework is unknown.

We aimed to investigate the psychological and motivational factors moderating changes in pain outcomes observed in chiropractic patients undertaking a novel telehealth self‐management programme. Specifically, we examined changes in pain intensity, pain cognitions and anxiety after participating in the programme. Further, we aimed to assess whether the latter influenced motivation and adherence to this novel way to deliver care. We hypothesized that anxiety and pain cognitions would impact motivation and adherence, moderating changes in pain intensity.

## 2 METHODS

A prospective pre–post observational study was performed in the setting of the Madrid College of Chiropractic Student Outpatient Clinic in San Lorenzo de El Escorial, Madrid, Spain (from hereon, the MCC Clinic). Ethical approval was received from the Madrid College of Chiropractic Research Ethics Committee (reference 010420). The current report follows the Strengthening the Reporting of Observational Studies in Epidemiology (STROBE) guidelines. The period of observation started on 4 April 2020 and was completed on 24 May of the same year, during the COVID‐19 nationwide lockdown. During this time period, citizens were confined to their homes except for essential business.

### 2.1 Patient recruitment

All participants were recruited from the database of the MCC Clinic by their assigned chiropractic intern, who contacted them via WhatsApp messenger (WhatsApp Inc). This messenger application was extensively used for telehealth before and during the pandemic in Spain (Ena, 2020; Hassan et al., 2020; Rodriguez‐Fortunez et al., 2019). The chiropractic intern provided in‐person care prior to the beginning of the study. Previous care always included an initial visit with a complete case history and physical examination, leading to a working and differential diagnosis process that allowed ruling out any red flags or contraindications to chiropractic care, which have been defined in clinical practice guidelines (Globe et al., 2016; Whalen et al., 2019). Therefore, the consultations were all follow‐up visits for a previously diagnosed pain condition, making them more suitable for telehealth (Reeves et al., 2021). Chiropractic patients ages 16 years and above with a working diagnosis of an acute or chronic pain condition were included in the study (see Table 1). A total of 250 patients were invited to participate in the programme and provide data for the study. A final sample of 208 patients initially accepted to participate. Four of them were under the age of 16, two did not provide written consent to use their data and one died due to COVID‐19 before providing follow‐up data and was therefore excluded from the cohort, leaving a final sample of 201 patients at baseline. In addition, five patients were lost to follow‐up; hence, the final cohort was made up of 196 participants.

**TABLE 1:** Baseline characteristics of the cohort participating in the self‐management strategies

Total sample, *n*	168
Gender, *n* (proportion %)	
Women	104 (62)
Men	64 (38)
Age (mean ± SD)	46.4 ± 16.8
Chief complaint, *n* (proportion %)	
Low back pain	54 (32)
Back pain	23 (14)
Neck pain	41 (24)
Headache	3 (2)
Upper extremity pain	15 (9)
Lower extremity pain	7 (4)
Maintenance care	25 (15)

### 2.2 Study protocol

**FIGURE 1:**
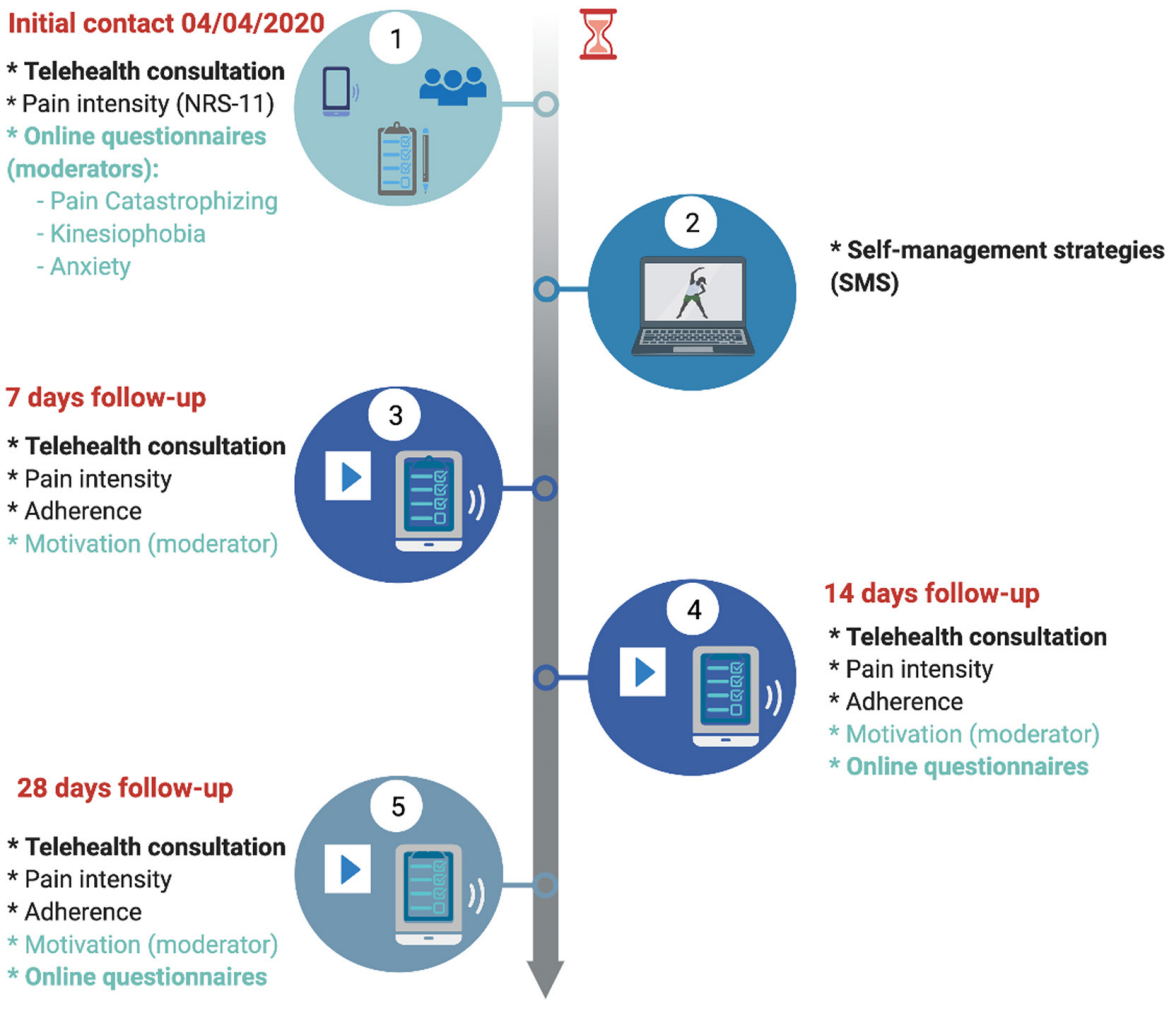
Study protocol and timeline. (1) Initial contact and examination consultation. (2) Provision of self‐management strategies in a video format. (3) First follow‐up consultation (day 7). (4) Second follow‐up consultation and re‐assessment (day 14). (5) Final re‐assessment (day 28).

This observational study investigated pre and post variables associated with the introduction of a novel telehealth programme based on self‐management strategies tailored to each individual patient. Patients were contacted for an initial teleconsultation via video (preferred mode of communication (Donaghy et al., 2019; Hammersley et al., 2019) and participation was offered at no cost. Patients provided verbal informed consent to participate and were assessed for their health status and chief complaint. This consultation included a brief clinical interview and, when necessary, a physical examination (to demonstrate symptom localization and reproduction). Immediately after this initial visit, they were required to complete an online self‐administered questionnaire designed in Google Forms (Google Inc.). On the following day, patients were contacted again by the same means and provided instruction on their tailored strategies for their chief complaint. These were delivered through pre‐recorded videos, to which patients had access until follow‐up at 14 and 28 days (see Figure 1).

### 2.3 Self‐management strategies

Chiropractic interns prepared an individualized video for each patient, according to their chief complaint and, when applicable, comorbidities or risk factors. Tailoring and personalization have been identified as enablers for the use, motivation and adherence to digital interventions for pain management (Fernandes et al., 2021; Svendsen et al., 2020). Videos were shared the day after the initial teleconsultation. They could be downloaded via the messenger application or watched online with a link to an online platform (YouTube or Google Drive, Google Inc.). So as to preserve confidentiality, videos were never addressed to a specific person’s name but rather kept anonymous.

The interns recorded themselves in a short video of ~5 min, explaining and demonstrating the strategies, namely a combination of movement, stretching and strengthening exercises. Relaxation or breathing exercises were also proposed for 43 of 168 patients (24%) and instructions on self‐massage to 28 patients (16%). Ergonomics, sleep, stress and anxiety management were less frequently used. All videos were previously supervised and approved by a clinical supervisor with 18 years of experience in the field. Examples of the videos are available in the original online platform (Presazzi, 2020) or as Videos S1–S3). Patients were requested to use the same strategies daily for at least 14 days until re‐assessment. At that point, a decision was made regarding continuing or discontinuing, adding to or modifying the strategies, depending on patients' needs and interests.

### 2.4 Primary outcome measure: pain intensity

The primary outcome measure was pain intensity, which was assessed using a numerical rating scale (NRS) from 0 (no pain) to 10 (worst pain imaginable). Pain intensity was assessed at the initial consultation and at two additional consultations 7 and 14 days after initiating the programme. The assessment after 7 days was used to identify any potential problems or questions concerning the implementation of the strategies. If participants continued care after the first re‐assessment, pain intensity was monitored again 28 days after baseline.

### 2.5 Secondary outcome measures and moderators

In addition to rating their pain intensity, a series of secondary outcomes were measured at different time points to be used as covariates and in order to identify potential moderators of response to the telehealth programme. First, patients were requested to evaluate retrospectively their adherence to the strategies during the previous 7 or 14 days, using a scale from 0 to 2, where 0 = ‘not using the strategy at all’, 1 = ‘using it inconsistently’ and 2 = ‘using it consistently’ (Nicholas et al., 2012). This was the only secondary variable that was not considered as a moderator in any model, as adherence is naturally considered a predictor rather than a moderator.

During the follow‐up visits, patients also assessed retrospectively their level of motivation to practice these strategies using a numerical rating scale from 0 (*no motivation at all*) to 10 (*maximum motivation*) in the follow‐up consultations after 7, 14 and, if applicable, 28 days (see Figure 1). Motivation is a key element influencing the use of self‐management strategies, whether face to face or remote (Söderlund & von Heideken Wågert, 2021; Svendsen et al., 2020).

Additionally, during the initial baseline assessment, and subsequently during the follow‐up re‐assessments at 14 and 28 days, patients completed an online questionnaire on their own. The first section of the questionnaire comprised demographic information (name of chiropractic intern, age, gender and number of cohabitants), after which consent to use the patient's responses was requested. If consent was not provided, the questionnaire was discontinued, and the complete patient data were excluded from the study. The second section included the following three scales: the Generalized Anxiety Disorder scale (GAD‐7) (García‐Campayo et al., 2010), the short version of the pain catastrophizing scale (PCS‐4) (Bot et al., 2014; Olmedilla Zafra et al., 2013) and the short version of the Tampa Scale of Kinesiophobia (TSK‐11) (Gómez‐Pérez et al., 2011).

The Spanish version of the GAD‐7 comprises 7 items rated each from 0 to 3, providing a minimum score of 0 and maximum of 21, where 10 is considered the cut‐off for diagnosing generalized anxiety. It has excellent internal consistency with a Cronbach's *ɑ* of 0.94 (García‐Campayo et al., 2010).

The short version of the PCS‐4 has not been validated in Spanish, though it correlates almost perfectly with the long version (*r* = 0.96) (Bot et al., 2014), which had been validated (Olmedilla Zafra et al., 2013). The PCS‐4 contains 4 items that are rated from 0 to 4 for a total score in pain catastrophizing ranging from 0 to 16.

The TSK has been analysed as a two‐, four‐ and five‐factor questionnaire, although recent data suggest that a two‐factor solution is preferable (Gómez‐Pérez et al., 2011). The Spanish version of the TSK‐11 uses 11 items scored from 1 to 4 for a total score for kinesiophobia or fear of movement ranging from 11 to 44. This version of the TSK showed the best reliability and validity with a two‐factor model: activity avoidance and harm. In this version, the ‘harm’ factor, also known as the somatic focus subscale, was the most useful at predicting pain outcomes (Gómez‐Pérez et al., 2011).

Finally, the follow‐up questionnaire added one item for patients to rate their satisfaction with the programme using a Likert scale from 1 to 5. This resulted in a total of 26 and 27 items initially and at follow‐up, respectively. All responses remained anonymous and were not accessible to the chiropractic interns.

### 2.6 Statistical analysis

Statistical analyses were performed with SPSS (v25 statistical package IBM SPSS Statistics) and the PROCESS module for SPSS (v3.3) (Hayes, 2017). All data are expressed as mean ± SD. A repeated measures ANOVA was performed with pain intensity ratings as the within‐subject factor and three levels (days 0, 7 and 14) to investigate the effect of treatment on pain intensity. Where the sphericity assumption was violated, a Greenhouse–Geisser adjustment was used. Significant effects were decomposed using planned contrasts to test a priori hypotheses and Bonferroni corrections were applied to determine the significance of two comparisons (day 0 vs day 7 and day 7 vs day 14). Further, in order to assess the influence of pain‐related cognitions on changes in pain intensity, the baseline score for all three questionnaires was included as a covariate in separate ANOVAs for analysis. Subsequently, a repeated measures ANOVA was repeated including a fourth level for day 28 to examine changes after day 14. Effect sizes are reported based on partial eta‐squared (*η* ^2^_*p*_). For exploratory reasons, the GAD‐7, PCS‐4 and TSK‐11 scores for days 0 and 14 were compared by means of three paired *t*‐tests. Posteriorly, *t*‐tests were repeated to compare the questionnaires’ scores between day 0 vs. day 28 and day 14 vs. day 28. A value of *p* < 0.05 was used as a threshold to determine statistical significance.

We expected to observe an impact of motivation and adherence on pain reports. However, research on psychological factors influencing performance rarely considers a direct relationship between two factors, independent of a third factor or group of factors. One of the most important revisions on the fear‐avoidance model for chronic pain took the role of motivation into account (Crombez et al., 2012). To explore the effect of pain cognitions and anxiety on the relationship between the patients’ motivation to pursue the recommendations, their adherence to them and changes in pain intensity, moderation analyses were used (Hayes, 2017). Amongst Hayes' models, model 1 suggests the involvement of a single most important factor in the relationship between a predictor and the outcome. This makes model 1 the most appropriate to explore this relationship. Furthermore, to assess how motivation influenced the relationship between adherence and pain intensity, we conducted another moderation analysis using again Hayes' model 1. Finally, the moderation models were tested with and without the inclusion of age and gender as covariates.

## 3 RESULTS

### 3.1 Demographics

A total of 196 patients provided baseline and follow‐up data for the first cut‐off point, 14 days after beginning the implementation of the strategies. Of these, 28 patients did not participate in the programme but still provided pre and post data that were not used for the analyses. A final cohort consisting of 168 participants was included on day 14. This comprised 25 patients who did not present pain at the specific time of recruitment and were categorized as ‘maintenance care’. All baseline and demographic characteristics of the final cohort are presented in Table 1.

A smaller number of patients prolonged their participation in the study beyond 14 days. In order to prevent the influence of different environmental factors on the measured outcomes, we restricted the study to the period of more stringent lockdown, when citizens in the Madrid region had significant restrictions in their mobility and access to in‐person healthcare was limited to emergency conditions. For this reason, only a subgroup of patients who had been recruited early in the study continued beyond the cut‐off of 14 days. For the second follow‐up 28 days after implementing the strategies, 40 patients provided partial data on pain intensity and 23 completed the follow‐up questionnaires.

### 3.2 Primary outcome measure: pain intensity

**FIGURE 2:**
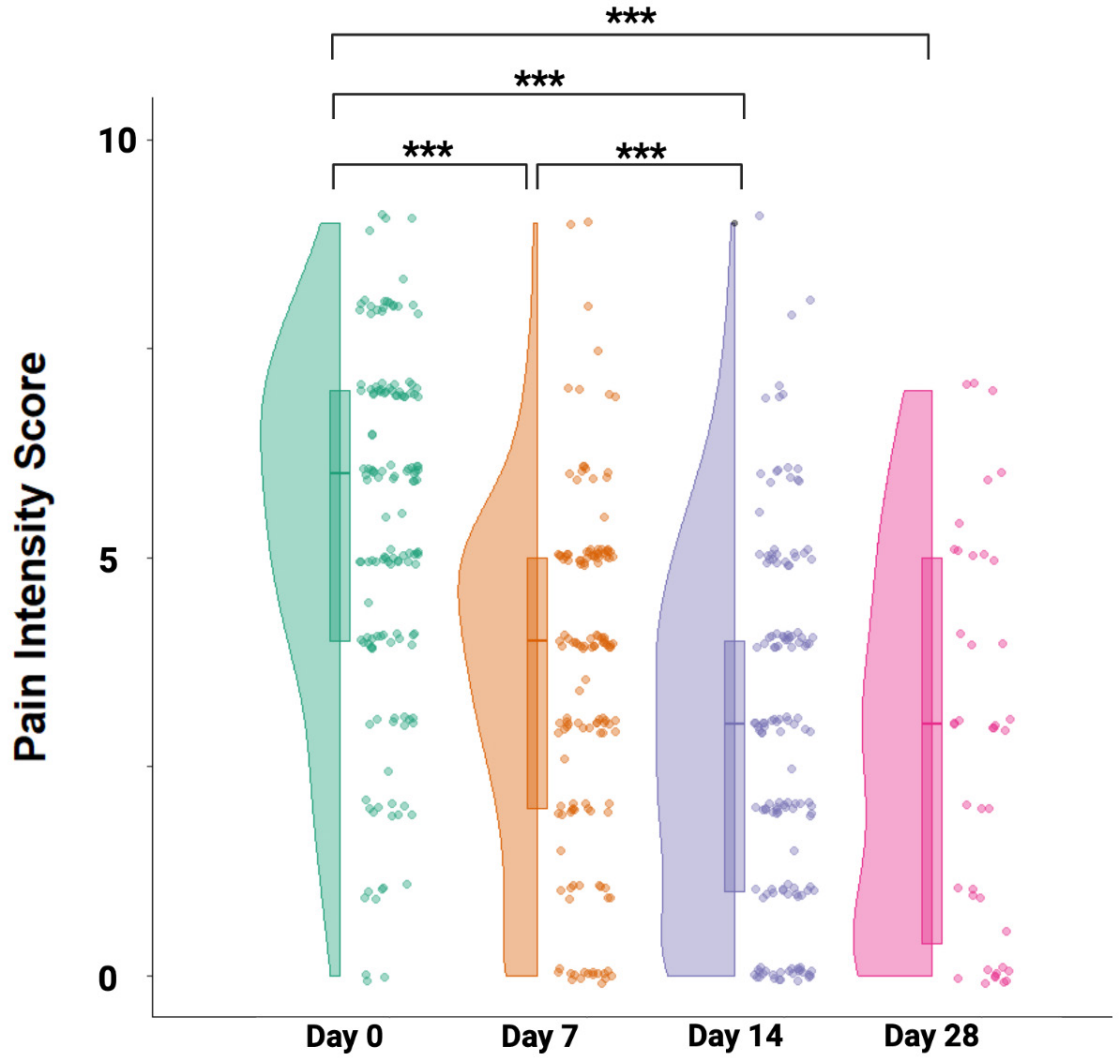
Evolution of pain intensity ratings. Violin plots represent pain intensity ratings on a numerical rating scale (0–10) on the days of the initial consultation (day 0) and follow‐up consultations on days 7, 14 and 28. Individual data points are represented by circles, boxplots illustrate median, 25th and 75th percentiles. ***p < 0.001.

Table 2 provides the means and standard deviation for all outcome measures during each follow‐up point. Our first hypothesis asserted that the introduction of the remote interventions would be associated with a reduction in pain intensity in our patients. The results of the ANOVA indicated a significant main effect of days: *F*_1.6,228.3_ = 110.03; *p* < 0.001; *η* ^2^_*p*_ = 0.43). Planned contrasts revealed that pain intensity on day 14 (mean: 2.9 ± 2.1) was significantly lower than on day 7 (mean: 3.56 ± 2.0, *p* < 0.001), which itself was significantly lower than baseline pain intensity (mean: 5.31 ± 2.1, *p* < 0.001) (see Figure 2 and Table 2).

**TABLE 2:** Baseline characteristics of the cohort participating in the self‐management strategies

	Baseline	7‐day follow‐up	14‐day follow‐up	28‐day follow‐up
Primary outcome				
Pain intensity (0–10), mean ± SD	5.3 ± 2.1	3.6 ± 2.0	2.9 ± 2.1	2.7 ± 2.3
Secondary outcomes and moderators				
Adherence (0–2), mean ± SD	—	1.6 ± 0.6	1.6 ± 0.6	1.6 ± 0.6
Motivation (0–10), mean ± SD	—	7.2 ± 2.4	7.1 ± 2.5	7.3 ± 2.6
PCS‐4 score (0–16), mean ± SD	5.0 ± 3.2	—	4.2 ± 3.2	5.2 ± 2.8
TSK‐11 score (11–44), mean ± SD	22.9 ± 5.5	—	21.7 ± 6.3	22.6 ± 7.3
GAD‐7 score (0–21), mean ± SD	4.9 ± 3.9	—	4.2 ± 3.9	4.3 ± 2.9
Satisfaction (1–5), mean ± SD	—	—	4.9 ± 0.4	4.8 ± 0.5

PCS–4 = Pain catastrophizing scale, short version; TSK–11 = Tampa scale of kinesiophobia, short version; GAD–7 = Generalized anxiety disorder scale

Results from the second repeated measures ANOVA examining changes at the 28‐day mark indicated a significant main effect of days: *F*_2.2,85.6_ = 40.06; *p* < 0.001; *η* ^2^_p_ = 0.51). Pain ratings on day 28 (2.75 ± 2.3) did not show any significant difference from those on day 14 (*p* = 0.95); however, they were still significantly lower than ratings at baseline (*p* < 0.001) (see Figure 2 and Table 2).

### 3.3 Secondary outcome measures: anxiety, pain catastrophizing and fear of movement

The scores for all secondary outcome measures at every follow‐up are displayed in Table 2. The questionnaires were reassessed after 14 days, showing a significant reduction in pain catastrophizing (*t*_163_ = 4.2, *p* < 0.001), kinesiophobia (*t*_163_ = 3.2, *p* = 0.001) and generalized anxiety questionnaire scores (*t*_163_ = 2.6, *p* = 0.01).

Three separate paired *t*‐tests were performed to compare the total scores at baseline and at 28 days. Reductions in pain catastrophizing (*t*_23_ = 2.3, *p* = 0.03) and generalized anxiety (*t*_23_ = 2.3, *p* = 0.03) remained significantly reduced at 28 days compared to baseline. However, this was not the case for kinesiophobia (*t*_23_ = 0.6, *p* = 0.5). No significant effect of the strategies was found when comparing all variables on days 14 and 28 (*p* = 0.88 for catastrophizing, 0.14 for anxiety and 0.85 for kinesiophobia).

### 3.4 Factors moderating changes in pain intensity

When including the baseline kinesiophobia scores as a covariate in the ANOVA, the main effect of days becomes insignificant (*F*_1.6,225.7_ = 3.1, *p* = 0.06, *η* ^2^_p_ = 0.02), suggesting that controlling for the effect of kinesiophobia, interventions were not associated with a reduction in pain. Interestingly, when kinesiophobia scores on day 14 were included in the model, changes in pain intensity were still significant (*F*_1.6,220.9_ = 7.2, *p* = 0.002, *η* ^2^_p_ = 0.05).

**FIGURE 3:**
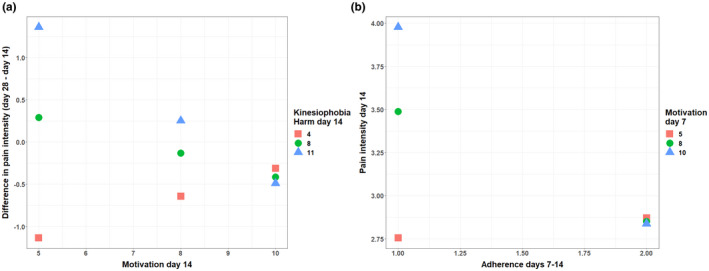
Moderation analyses. (a) Moderation by the *harm* subscale of the TSK‐11 of the relationship between motivation at day 14 and changes in pain intensity between days 14 and 28. Participants with low levels of motivation (5/10) and high levels of harm beliefs (triangles) had increased pain on day 28, whereas those with low motivation and low harm beliefs (squares) had reduced pain on day 28. (b) Moderation by motivation on day 7 of the relationship between adherence to the programme between days 7 and 14 and pain reported on day 14. Participants with high motivation 7 days after the beginning of the programme (triangles) but low adherence to the programme in the next 7 days (score of 1 out of 2), had higher pain intensity than those who had low motivation (squares) and adherence. When high adherence was reported, motivation did not influence pain intensity.

Accordingly, the kinesiophobia score on day 14 showed a trend towards significance when included as a moderator in the relationship between motivation on day 14 and change in pain intensity between days 14 and 28 (Δ*R* ^2^ = 0.07, *F*_1,36_ = 3.75, *p* = 0.06). The total kinesiophobia score was replaced with the *harm* subscale, which significantly moderated the relationship between motivation and pain reduction (Δ*R* ^2^ = 0.13, *F*_1,36_ = 7.8, *p* = 0.009). As shown in Figure 3a, *harm* scores moderated pain reductions only for participants with low levels of motivation.

We were also interested in understanding how motivation and adherence influenced pain reductions. A moderation analysis showed that the motivation reported on day 7 (which was the first report) moderated the relationship between the use of service onwards (between days 7 and 14) and pain intensity on day 14 (Δ*R* ^2^ = 0.05, *F*_1,135_ = 6.5, *p* = 0.01). As shown in Figure 3b, this moderation effect was observed for those who adhered less to the programme.

When assessing the influence of pain and gender in the model by including them as covariates in the moderation analyses, the overall effect remains unchanged, suggesting a lack of interaction between moderating factors, age and gender.

## 4 DISCUSSION

The present study assessed the moderators of response to a novel telehealth self‐management programme for chiropractic patients during COVID‐19 lockdown in Spain. Fear of movement arose as an important factor influencing changes in pain intensity and moderating the relationship between motivation and pain reductions. Patients with low motivation experienced pain increases when a higher degree of ‘harm beliefs’ were present, yet low levels of these beliefs led to pain reductions. Such a difference disappeared when individuals were highly motivated. Motivation moderated the relationship between adherence and pain intensity. If adherence was low, high motivation was actually associated with higher pain intensity. However, when high adherence was reported, differences in motivation did not influence pain.

Patients participating in the telehealth programme reported high levels of satisfaction (4.9/5 ± 0.4) 2 weeks after initiating the programme. These are comparable to the levels reported with in‐person care during the pandemic in Spain (Gevers‐Montoro et al., 2022) but largely exceed satisfaction amongst patients receiving other telehealth programmes (Carrillo‐de‐la‐Pena et al., 2021). Decreases in pain intensity with a large effect size (*η* ^2^_*p*_ = 0.43) were also reported. These could be expected due to the natural course of some pain conditions. Moreover, the lack of a control group does not allow for inferences regarding the effectiveness. Changes in pain intensity were not found to be significant when controlling for baseline levels of kinesiophobia. This indicates that fear of movement could help predict the patients’ response. Maladaptive pain cognitions and beliefs, including kinesiophobia, are associated with increased pain and poor adjustment to pain (Keefe et al., 2004; Luque‐Suarez et al., 2019). In turn, kinesiophobia may be reduced by exercise programmes for low back pain patients (Hanel et al., 2020; Martinez‐Calderon et al., 2020). In our cohort, exposure to exercise strategies may have influenced the degree of fear of movement, but also catastrophizing and anxiety. Similar results were previously reported 3 weeks after teaching a comprehensive self‐management programme containing stretching exercises for patients with chronic pain (Nicholas et al., 2012).

In the context of COVID‐19, self‐management is one of the best options available for pain relief. The strategies implemented in the present study were predominantly active interventions, including movement and physical activity. Current best practice recommendations emphasize the use of active strategies and self‐management for musculoskeletal pain (Kongsted et al., 2021; Lin et al., 2020). However, adherence is essential for better outcomes from active care (Nicholas et al., 2012; Nicholas & Blyth, 2016). Motivation has been proposed as an important and often lacking component influencing participation in pain self‐management programmes (Ackerman et al., 2013; Söderlund & von Heideken Wågert, 2021), including those provided remotely (Svendsen et al., 2020). We found that the relationship between motivation and changes in pain intensity after the first 2 weeks was marginally moderated by the TSK‐11 scores (*p* = 0.06). This is consistent with data showing that kinesiophobia moderates low back pain treatment efficacy (Wertli et al., 2014).

The *harm* factor emerged as a stronger moderator influencing pain intensity, though only for patients with low levels of motivation. For these patients, high scores in this subscale were associated with increased pain on day 14, whereas for those with low *harm* scores and the same motivation, pain decreased (see Figure 3a). This factor, which reflects the belief in the presence of a serious underlying pathology (Goubert et al., 2004), was also found to reduce the likelihood of benefitting from hands‐on care during the pandemic (Gevers‐Montoro et al., 2022). The present study expands on these findings by providing evidence that these beliefs, when combined with low motivation to actively engage in exercise whilst in home confinement, could lead to worsening of pain symptoms.

We further speculated that the relationship between the degree of adherence to the programme and pain intensity could be explained by motivation. Lack of motivation and interest was identified as key elements influencing adherence to self‐management (Ackerman et al., 2013). Yet, motivation is essential in driving the behavioural changes necessary for active self‐care (Söderlund & von Heideken Wågert, 2021). Even if motivation is present, it does not always lead to participation due to unidentified factors limiting the necessary behavioural changes (Miller et al., 2020). Our findings suggest that motivation levels moderate the relationship between adherence and pain intensity. As expected, high adherence influenced pain intensity, and this was independent of motivation (see Figure 3b). Previous data suggest that adherence can predict improvement in pain outcomes, even after controlling for fear‐avoidance beliefs (Nicholas et al., 2012).

High motivation in those who adhered less to self‐management during the second week was associated with higher pain intensity. It is possible that some of the necessary motivation components for adherence were lacking in this subgroup. These have been described in the literature as readiness to change, self‐monitoring and goal setting (Söderlund & von Heideken Wågert, 2021). Beyond motivation, capability and opportunity are also considered enablers of behavioural change. In the context of the pandemic and home confinement, some key elements of opportunity (e.g. environmental triggers, social support) may be particularly scant for a subset of patients. However, these components were not assessed in the present study.

It could be argued that high motivation without capability or opportunity may lead to unmet expectations of self‐efficacy and clinical outcomes (Jensen et al., 2003). Outcome and self‐efficacy beliefs (expectancies) impact behaviour, even more than the actual consequences of behaviour. Hence, a patient with high motivation but poor self‐efficacy (capability) or support (opportunity) (Fernandes et al., 2021; Söderlund & von Heideken Wågert, 2021) might discontinue the programme if quick relief is not attained. This is consistent with a moderating role of kinesiophobia, which often correlates negatively with self‐efficacy (de Moraes Vieira et al., 2014; Ferrari et al., 2016; Jochimsen et al., 2021). However, these moderators need to be further explored in a separate study.

Additional factors that may be considered to have influenced adherence and were not measured are quantity, quality, usability or enjoyment of the digital content (Svendsen et al., 2020; Whiteley et al., 2006). Clinician‐related factors may also act as potential barriers for delivering care remotely. Beliefs of inferior effectiveness compared to face‐to‐face care, physical opportunity factors (for both clinician and patients) and lack of proper training may hinder the routine use of telehealth services (Malliaras et al., 2021).

Despite the novelty of this study, there are limitations that need to be considered when interpreting the results. First, the interpretation of the results may be limited to a small population of chiropractic patients from a teaching institution. Additionally, variables were self‐reported. This may be particularly relevant for adherence, which could not be monitored objectively. Furthermore, to preserve the same conditions of strict home confinement throughout the study, patient recruitment was time limited. The announcement of lockdown easing in May meant that in‐person care would be made available, altering the study's environmental conditions. This explains why, despite high levels of satisfaction, participation was largely discontinued after 2 weeks. Most importantly, we were unable to recruit a control group. This is the main limitation of the study, which prevents the inference of causal relationships between the intervention and the observed effects.

An alarming lack of initiatives to mitigate the effects of social distancing in patients with pain has been identified (Carrillo‐de‐la‐Pena et al., 2021). Adapting a traditionally hands‐on healthcare service such as chiropractic towards a remotely delivered self‐management programme poses the challenge of identifying those patients who are most likely to respond to telehealth and those who are not. The present study findings suggest that, in order to promote adherence, telehealth self‐management programmes should be personalized by taking into consideration pain cognitions and motivation, but also possibly self‐efficacy, capability and opportunity. Moreover, for patients with high levels of fear of movement, increased motivation can be a determinant factor for pain reduction. However, if motivation is increased, adherence should be monitored to achieve significant pain relief. Further investigation is needed to reduce barriers and facilitate the role of clinicians as self‐management enablers during social distancing.

## AUTHOR CONTRIBUTIONS

CG‐M, ZD, AOdM and AK contributed to conceive and plan the overall study design. The questionnaires were developed by CG‐M and ZD. CG‐M collected the data. AK performed the statistical analyses. EG contributed to the analysis and plotting of the results. AF contributed to interpreting the data and results and elaborating the discussion. CG‐M, ZD, EL and AF contributed to manuscript writing and editing. All the authors reviewed the manuscript, and the final version was edited and approved by AK. The team met regularly and contributed as a whole to discussions of the data.

## CONFLICT OF INTEREST

This study was fully self‐funded, and the authors received no specific funding for this work. All authors declare no financial relationships with any organizations that might have an interest in the submitted work and no other relationships or activities that could appear to have influenced the submitted work.

## Supporting information

Demonstration Videos

Demonstration Videos

Demonstration Videos
